# Impact of shift durations on sleep, fatigue, and wellness among neonatologists: a cross-sectional survey analysis

**DOI:** 10.1038/s41372-025-02310-4

**Published:** 2025-04-25

**Authors:** Ryan M. McAdams, Renate Savich, Patrick J. McNamara, Lily Lou, Jens C. Eickhoff, Satyan Lakshminrusimha

**Affiliations:** 1 Department of Pediatrics, University of Wisconsin School of Medicine and Public Health, Madison, WI USA; 2 Department of Pediatrics, University of New Mexico Health Sciences Center, Albuquerque, NM USA; 3 Department of Pediatrics, University of Iowa Carver College of Medicine, Iowa City, IA USA; 4 Department of Pediatrics, University of Illinois Chicago, Chicago, IL USA; 5 Department of Pediatrics, University of California Davis Children’s Hospital, Sacramento, CA USA

**Keywords:** Quality of life, Health occupations

## Abstract

**Objective:**

To assess the effects of shift durations on sleep, fatigue, and wellness among U.S. neonatologists in diverse settings.

**Methods:**

A cross-sectional survey of U.S. neonatologists yielded 810 responses from 4400 recipients. Statistical analyses included ANOVA, logistic regression, and cluster analysis.

**Results:**

Younger neonatologists (<35 years) reported the highest fatigue levels, with females more affected than males (*p* = 0.0185). Male neonatologists were less likely than females (OR 0.55, *p* = 0.0013), and those in university settings more likely than non-university settings (OR 1.43, *p* = 0.0389), to report adverse effects of shifts >16 h. Cluster analysis identified three fatigue patterns, with the most severe among younger neonatologists working shifts >16 h.

**Conclusion:**

Long shifts (>16 h) negatively affect neonatologists’ wellness, particularly younger and female clinicians. With most neonatal-perinatal medicine fellows being female, policies addressing shift duration are needed.

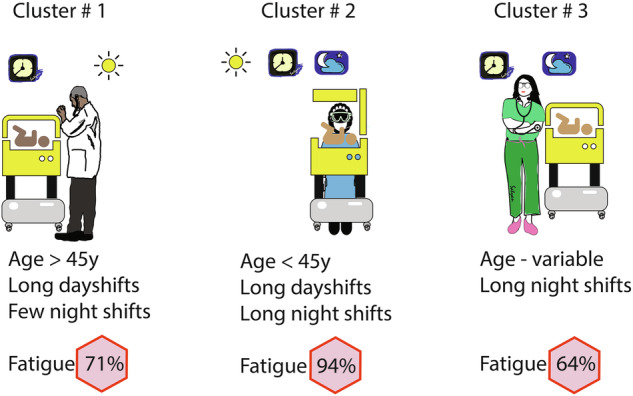

## Introduction

Neonatologists provide essential care to critically ill newborns in neonatal intensive care units (NICUs), often working long and irregular shifts. In this study, long shifts are defined as those lasting 16 h or more, a threshold supported by evidence linking shift durations beyond this point to increased rates of serious medical errors, attentional failures, and provider harm [1]. Research demonstrates that after 16 consecutive hours of wakefulness, measurable deficits in attention and executive function emerge, with psychomotor performance impairments comparable to those observed at legally intoxicating blood alcohol levels [2]. Although the term ‘extended shifts’ is variably defined in the literature, sometimes referring to shifts of 24 h or longer [3–5], evidence identifies 16 h as a critical inflection point for provider fatigue, medical error, and patient safety [6]. A systematic review of intervention studies demonstrated that resident physicians working shifts exceeding 16 h experienced more attentional failures, impaired neurobehavioral performance, and committed significantly more serious medical errors compared to those working schedules limited to 16 consecutive hours [7].

Neonatologists frequently navigate irregular shift patterns, alternating between day and night duties, further complicating their sleep-wake cycles and recovery times [6, 8, 9]. Studies have demonstrated that shifts >24 h impair neurobehavioral performance and contribute to increased attentional failures among resident physicians, highlighting similar risks for neonatologists [6, 10]. Despite this growing body of evidence, there is limited research specifically examining the effects of shift duration on attending neonatologists, whose roles and responsibilities differ from those of resident physicians.

The demands of shifts >24 h and irregular shifts have profound implications for clinician performance and wellness. Sleep deprivation is a well-documented factor for cognitive impairment, manifesting as reduced executive functioning, increased impulsivity, and slower cognitive processing times, which can directly affect clinical decision-making and performance [11]. Furthermore, sleep deprivation has been associated with significant safety concerns, as it not only increases the likelihood of medical errors but also affects communication and teamwork in high-stakes environments [12]. Prolonged and irregular shifts also detract from overall well-being, contributing to mental health challenges such as depression and anxiety. Kalmbach et al. found that sleep deprivation during medical training significantly increases the risk of developing depression, which can further exacerbate the likelihood of medical errors [13].

Although the risks of sleep deprivation and extended shifts are well established, questions remain about the optimal shift lengths for attending neonatologists to support both patient safety and clinician wellness. Existing studies on resident physicians have shown that reducing shift durations improves alertness and reduces the risk of medical errors, but data specific to NICU providers are sparse [14]. With 76.7% of first-year fellows in neonatal-perinatal medicine being female, it is critical to understand the interaction between shift duration, physician gender, provider’s age, and fatigue [15].

In response to growing concerns about the impact of prolonged shifts on neonatologists [16], we conducted a nationwide survey to assess how shift durations influence sleep patterns, fatigue levels, and perceived well-being among this specialized group. Our survey targeted neonatologists across various practice settings to capture a comprehensive view of their experiences and opinions regarding shift work.

This study provides data-driven insights into the relationship between shift lengths and the perceived health and safety of neonatologists. By examining demographic predictors including gender, age and work patterns, this research aims to inform policy changes that promote sustainable and healthy work practices in the NICU.

## Methods

### Study design and participants

This cross-sectional survey study was conducted to assess the impact of shift durations on the sleep safety and wellness of neonatologists. The target population comprised US neonatologists working in academic or non-academic NICUs. The survey utilized a non-probability sampling method, with an open survey link distributed through the Section on Neonatal-Perinatal Medicine (SoNPM) listserv. This listserv includes approximately 4400 separate emails, but due to multiple emails for an individual, as well as non-practicing neonatologists and non-neonatologists on the listserv, it is unknown precisely how many individual practicing neonatologists are on the listserv. The listserv includes approximately 2/3 of all board-certified neonatologists. The listserv members comprise a diverse group of neonatal-perinatal healthcare professionals, including over 400 trainees, 60 neonatal nurse practitioners, and 100 pediatricians. The SoNPM leadership reviewed and approved the survey, ensuring its alignment with their mission to improve maternal, fetal, and neonatal health while protecting member privacy. Participation was voluntary, and the survey was accessible from April 22 to May 6, 2024. The study received approval from the Institutional Review Board of the University of Wisconsin-Madison.

### Survey instrument

The survey instrument was developed collaboratively by the research team and the University of Wisconsin Survey Center. It underwent technical review and was programmed in Qualtrics (Qualtrics, Seattle WA) for web administration. The survey was available only in English and consisted primarily of multiple-choice questions, including Likert-scale, yes/no, and numeric response questions. Due to a programming error, three questions were omitted for the first 227 respondents: (1) ‘In the past month, about how many times did you get less than 7 h of sleep in a 24-hour period?’ (2) ‘In the past 6 months, how often did your fatigue or lack of sleep contribute to significant medical errors or near misses?’ and (3) ‘Do you have a sleep disorder, such as insomnia?’ This issue was corrected, and the remaining 583 respondents completed the full survey. Analyses involving these variables were limited to the subset of 583 respondents to avoid introducing bias. The survey was designed to allow respondents to skip questions if desired, ensuring data integrity while respecting participant autonomy.

### Data collection

Data collection was facilitated through two email invitations sent via the listserv of neonatologists. These emails included a brief description of the study, an invitation to participate, and a link to the online survey (see Appendix for the survey tool). Reminder emails were sent to maximize participation. The survey was designed to be completed in one sitting, and participants could not return to the survey once they exited. Data were collected using Qualtrics software, which ensured confidentiality and secure storage in compliance with institutional policies.

### Measures

The survey comprised 20 questions addressing various aspects of NICU shift durations and their perceived impacts on sleep, safety, and wellness. Long shifts were defined as those exceeding 16 h. This threshold was selected based on survey questions asking participants to evaluate the effects of shifts of this duration on their well-being and patient safety. Key variables included the frequency of fatigue during NICU shifts, measured on a Likert scale, and the typical duration of both day and night shifts. Self-reported fatigue levels were assessed using a 5-point Likert scale, where participants were asked, ‘In the past month, how often did you feel fatigued or sleepy during your work shifts in the NICU?’ Responses ranged from 1 (‘Never’) to 5 (‘Extremely often’). Higher scores indicated more frequent experiences of fatigue or sleepiness during work shifts. Respondents were also queried about their longest shift duration. Opinions were gathered on the optimal shift lengths for patient safety and neonatologist wellness. Additionally, the survey assessed the frequency of sleep less than seven hours within a 24-hour period and the impact of fatigue on medical errors or near misses. It also recorded the additional hours dedicated to NICU-related tasks outside scheduled shifts, as well as the use of sick leave and vacation time. Demographic information collected included age, gender, years of practice, practice setting, NICU level, and the presence of sleep disorders.

### Statistical analysis

Descriptive statistics were computed to summarize the demographic characteristics of the respondents and their responses to the survey questions. Fatigue levels were compared between demographic characteristic groups (age categories, sex) using analysis of variance (ANOVA). Model assumptions were verified by examining residual plots. Additionally, two-way ANOVA was used to examine interaction effects between variables, such as the combined effect of age and gender on fatigue frequency. Logistic regression was used to evaluate the associations between demographic characteristics and the presence of a sleep disorder. Latent class analysis was utilized to identify distinct profile clusters (classes) of responders based on fatigue levels, hours worked at day and night shifts, hours to perform NICU shifts. The number of profile clusters was determined by conducting an iterative analysis approach, starting with a model with 2 (k) classes. The Vong-Lo-Mendell-Rubin likelihood ratio test was used to compare the model with k classes to a model with k – 1 classes. A significant p value (p < 0.05) indicates that the model with k classes provides a better model fit than the corresponding model with only k – 1 classes. This analysis was repeated until there was an indication (p > 0.05) of improvement in model fit. All reported p values are two-sided, and p < 0.05 was used to define statistical significance. Statistical analyses were conducted using SAS software (SAS Institute, Cary NC), version 9.4 and Mplus software (Muthén & Muthén, Los Angeles, CA, USA) version 8.

### Ethical considerations

Participation in the study was voluntary, and informed consent was obtained from all respondents. The survey was anonymous, and data confidentiality was maintained according to the University of Wisconsin’s policies. Identifiable or sensitive information was stored separately from other study data to minimize the risk of confidentiality breaches. The study posed minimal risks to participants, primarily involving the potential for confidentiality breaches, which were mitigated through secure data storage and handling procedures.

## Results

A total of 810 neonatologists responded to this nationwide survey. Due to the unknown total number of surveyed individuals actively engaged in clinical neonatology, the exact response rate could not be calculated. The demographic characteristics of the respondents included a median age of 46 years (range: 29–79) and a median of 12 years practicing as neonatologists (range: 0–48). Of the 810 respondents, 71% were female, 29% were male, and 1 respondent was non-binary. Most respondents worked in university settings (58%), with a significant portion reporting work in both Level 3 (81%) and Level 4 (70%) NICUs. Additionally, 15% of respondents reported having a sleep disorder or insomnia (Table 1).

**Table 1:** Demographic characteristics of respondents.

Characteristic	N	% or Median (Range)	
Total Respondents	810	100%	
Age (years)		46 (29–79)	
Years Practicing as Neonatologist		12 (0–48)	
Gender			
- Male	236	29%	
- Female	568	71%	
- Non-Binary	1	0%	
Practice Setting			
- University	464	58%	
- Non-University	342	42%	
NICU Level Work Exposure			
- Level 2	333	45%	
- Level 3	629	81%	
- Level 4	475	70%	
Reported Sleep Disorder/Insomnia	86	15%	
Questions 10, 11, and 20 Respondents			
- Completed	583	72%	
- Missed	227		28%

Fatigue levels varied significantly by age and gender. Younger neonatologists (25–34 years) reported the highest self-reported fatigue level (3.2, SD 0.8), while those aged ≥65 years reported the lowest (2.8, SD 0.9). Female neonatologists experienced significantly higher fatigue levels compared to males (mean score: 2.9 vs. 2.6, *p* = 0.0185). Logistic regression indicated that female neonatologists and those working in university settings were more likely to report that long shifts (>16 h) negatively impacted their well-being (OR 0.55, *p* = 0.0013 for males when compared to females; OR 1.43, *p* = 0.0389 for university settings when compared to non-university setting). Detailed fatigue levels and well-being impacts are presented in Table 2.

**Table 2:** Fatigue levels and well-being impact by age, practice setting and gender.

Age group (years)	N	^a^Self-reported mean fatigue level (SD)	% Reporting negative impact on well-being	Odds ratio for negative impact (95% CI)^b^	p value
Age Group (years)					
25–34	81	3.2 (0.8)	82%	2.00 (1.04–3.84)	0.1042
35–44	296	3.2 (0.8)	73%	1.16 (0.69–1.94)	0.6107
45–54	210	3.1 (0.8)	72%	1.14 (0.66–1.95)	0.6702
55–64	139	3.1 (0.8)	77%	1.44 (0.77–2.70)	0.2558
≥65	77	2.8 (0.9)	70%		
Practice Setting					
University	462	3.1 (0.8)	78%	1.43 (1.02–2.02)	0.0389
Non-University	338	3.1 (0.9)	72%		
Gender					
Male	236	2.6 (1.2)	69%	0.55 (0.37–0.83)	0.0013
Female	568	2.9 (1.1)	80%		

*SD* standard deviation, *CI* confidence interval.

^a^Responses ranged from 1 (‘Never’) to 5 (‘Extremely often’). Higher scores indicated more frequent experiences of fatigue or sleepiness during work shifts.

^b^Based on multivariate model with age group, practice setting, and gender as predictors.

Respondents were asked to rate the extent to which shift durations exceeding 16 h increased risks to patient safety on a 5-point Likert scale (1 = “Not at all” to 5 = “A great deal”). Female respondents reported a higher perceived risk compared to males, with mean scores of 2.9 (SD 1.1) and 2.6 (SD 1.2), respectively (*p* = 0.0185).

Latent class analysis revealed three distinct response patterns among neonatologists. Cluster 1 included older providers (mostly aged >45 years) who predominantly worked long day shifts but not long night shifts. This group reported moderate levels of fatigue (71%) and a higher likelihood of working over 10 h during the day (100%). Cluster 2 consisted of younger providers (mostly aged ≤45 years) who experienced high fatigue levels (94%) and worked long shifts both during the day (57%) and night (76%), with a significant proportion working shifts longer than 25 h (100%). Cluster 3 comprised respondents who did not work long day shifts but were more likely to work long night shifts (62%), and this group had moderate levels of fatigue (64%). These clusters reveal different patterns of shift work and fatigue among neonatologists, with younger providers and those working longer shifts reporting higher fatigue levels (Table 3).

**Table 3:** Cluster Analysis of Response Patterns.

Cluster	N	Feeling Fatigue/Sleepy (Sometimes/Very often/Extremely often) (%)	Hours Worked (Day Shift ≥ 10) (%)	Hours Worked (Night Shift ≥ 16) (%)	Hours (Longest Shift ≥ 25) (%)	Hours (NICU Tasks ≥ 12) (%)	Age > 45 years (%)
Cluster 1	284	71%	100%	31%	26%	55%	70%
Cluster 2	372	94%	57%	76%	100%	50%	36%
Cluster 3	154	64%	0%	62%	5%	39%	49%

## Discussion

Our findings demonstrate that shift durations significantly impact the sleep, fatigue, and well-being of neonatologists, with critical implications for clinician wellness and patient safety. Long shifts (>16 h) were associated with increased fatigue across all demographic groups, with younger neonatologists and female physicians reporting the highest levels. These trends align with prior studies identifying age and gender as significant predictors of physician fatigue [17, 18].

Female neonatologists, in particular, reported disproportionately high fatigue, consistent with research showing that women physicians experience greater challenges in work-life integration and increased domestic responsibilities [19–21]. Studies indicate that women physicians, even when working similar clinical hours, spend approximately 8.5 more hours per week on household and childcare duties, contributing to greater fatigue and lower satisfaction with work-life balance [19].

Despite efforts to mitigate fatigue through reduced work hours, persistent disparities demonstrate the need for structural changes, as cultural and workplace norms often reinforce these pressures and limit opportunities for recovery [20, 22]. Studies also indicate that women physicians experience higher levels of emotional exhaustion compared to their male colleagues, which may be compounded by societal expectations and work-life balance pressures [23]. Our study findings suggest that female neonatologists are more likely to associate longer shifts with increased patient safety risks, suggesting potential differences in how fatigue-related risks are perceived across genders. While female neonatologists report disproportionately high fatigue, our findings also indicate that fatigue is a pervasive issue across all neonatologists, regardless of gender, in the high-stakes NICU environment. Addressing these disparities is essential not only for clinician wellness but also for patient safety, as fatigue in high-acuity settings such as the NICU can impair decision-making and increase the risk of medical errors.

Fatigue is a well-documented contributor to impaired cognitive performance, increased medical errors, and higher malpractice risks [9, 10]. Extended shifts disrupt circadian rhythms, exacerbate sleep deprivation, and reduce alertness, particularly during night shifts, posing substantial risks in high-stakes NICU environments [11, 24]. These effects are especially concerning in the NICU, where continuous vigilance and split-second decision-making are critical for patient outcomes. Failure to address widespread fatigue may not only increase medical errors and malpractice risks but also contribute to physician attrition, further exacerbating workforce shortages in neonatal care. Similar to aviation safety protocols that manage pilot fatigue, structured interventions are essential to minimize clinician fatigue-related risks and optimize decision-making [12, 25].

Cluster analysis in our study revealed distinct fatigue patterns, with younger neonatologists reporting significantly higher levels of fatigue during both day and night shifts. Similar findings were reported by LaFaver et al. [17], who noted that burnout and fatigue were more prevalent among younger neurologists compared to their older counterparts. Younger clinicians, trained under duty hour restrictions and with greater emphasis on physician wellness during residency, may be more attuned to the impact of fatigue on their performance [26]. In contrast, clinicians who trained before duty hour limits—during an era of longer shifts and more consecutive work weeks—may have acclimatized and adjusted to higher levels of fatigue, potentially influencing how they perceive or report fatigue symptoms. Additionally, our study did not account for whether younger neonatologists worked disproportionately more night shifts, which could have contributed to the observed fatigue levels. Older neonatologists in our cohort typically worked day shifts, potentially reducing their exposure to fatigue associated with night work. Moreover, in university-based practices, competing demands for clinical productivity, teaching, research, and administrative duties, compounded by a lack of standardized clinical full-time equivalent definitions, may contribute to higher fatigue among neonatologists [27]. These findings highlight the need for further exploration of work patterns and targeted mentorship and wellness programs to support early-career neonatologists.

Neonatologists trained in different eras have experienced varying duty-hour structures, but the fundamental physiological need for sleep remains unchanged. While those trained before duty-hour restrictions may have adapted their workflows to long shifts, this does not equate to an increased resistance to fatigue. As the workforce evolves, with a growing proportion of neonatologists trained under restricted work hours, it is important to recognize that fatigue is shaped by multiple factors, including age, individual physiology, workload, and personal responsibilities. Workforce adaptations may also influence how neonatologists experience fatigue over time. Older neonatologists or those with caregiving responsibilities may transition into part-time roles or modify shift structures to mitigate fatigue. Future research should explore how these evolving work patterns impact long-term workforce sustainability and clinician well-being.

The long-term health risks associated with extended shifts extend beyond fatigue and include cardiovascular disease, metabolic disorders, and mental health consequences [28]. Shift work has been shown to impair metabolism, increase cardiovascular disease risk, and disrupt circadian function, raising concerns about chronic health deterioration among neonatologists [28]. Additionally, fatigue-related cognitive impairment affects executive function, memory, and processing speed, further compromising performance [29].

Our study has several limitations. The cross-sectional design prevents causal inference between shift duration and fatigue levels. Additionally, the use of a non-probability sampling method may have introduced response bias, limiting generalizability to all U.S. neonatologists. The self-reported nature of our survey also introduces potential bias, as subjective assessments of fatigue and sleep patterns may not fully align with objective sleep measures [6].

Furthermore, incomplete responses from some participants prevented a full analysis of certain variables related to sleep patterns, fatigue-related errors, and sleep disorders. Our survey did not account for important fatigue-modifying factors, including pregnancy status, caregiving responsibilities, access to childcare, differences between day and night shifts, and distinctions between in-hospital shifts and home call, all of which can influence sleep patterns and fatigue-related outcomes. These limitations may underestimate the extent of gender disparities in fatigue. Additionally, we did not assess patient acuity, a key factor influencing workload intensity and fatigue.

Our study also did not capture the diversity in work models among neonatologists, including differences in rural vs. urban settings, availability of advanced practice providers, and variations in staffing structures. Further studies should explore differences in workload intensity, practice settings, and staffing models to develop tailored interventions that address fatigue-related risks in diverse NICU environments. Future research using actigraphy or polysomnography could provide more objective measures of sleep quality and duration among neonatologists [6]. Finally, the absence of open-ended response options may have constrained participants from providing detailed insights into their experiences with fatigue.

Efforts to optimize shift structures must balance provider well-being with continuity of patient care. Standardizing evidence-based fatigue mitigation strategies, including protected sleep periods and structured breaks, is essential for supporting the workforce and optimizing patient care [30]. Fatigue mitigation strategies must address both individual and systemic factors to ensure sustainable work practices in neonatology. While shorter shifts have been proposed to reduce fatigue, they present logistical challenges, including more frequent handoffs, potential disruptions in continuity of care, and financial implications in non-university settings where productivity-based compensation models exist. A balanced approach should consider clinician wellness, operational feasibility, and patient safety when implementing shift modifications. Evidence-based guidelines for NICU scheduling must account for these complexities.

Long shifts that include home call introduce additional variability in fatigue impact, depending on factors such as NICU acuity, availability of advanced practice providers or fellows, and personal responsibilities outside of work. In some systems, home call receives lower full-time equivalent credit, potentially increasing total work hours and contributing to chronic fatigue and burnout. Addressing fatigue-related risks in both academic and non-academic settings will require carefully structured policies that define work-hour expectations and provide adequate recovery time.

Given the well-documented risks associated with prolonged shifts, we recommend capping work shifts at no longer than 24 h. Studies have demonstrated that 24-hour on-call shifts significantly impair cognitive performance, reduce concentration-endurance, and increase memory deficits, decision-making errors, and processing delays [1, 10, 29, 31]. Additionally, extended shifts have been linked to higher rates of medical errors, injuries, and attentional failures [1, 25]. Recent neonatology staffing consensus recommendations similarly underscore the lack of ethical or practical justification for extended in-hospital shifts exceeding 24 h, particularly given their negative impact on clinician well-being and patient safety [32]. While a 16-hour shift cap may be an ideal long-term goal, a 24-hour limit is an essential first step toward safer work environments and reduced clinician fatigue.

Although concerns about increased handoffs exist, evidence from emergency medicine and pediatric critical care supports the feasibility of shorter shifts. Studies show that limiting shifts to ≤16 h reduces resident fatigue and improves sleep [33, 34]. While one study involving pediatric resident physicians during their intensive care unit rotations reported increased medical errors after eliminating extended shifts, this was confounded by workload, which, when adjusted, showed no significant impact on patient safety [3]. These findings underscore that safe care depends on both shift duration and effective workload management.

Beyond shift length modifications, a sustainable approach to clinician wellness requires cultural and systemic change. Peer support programs, structured fatigue education, and institutional wellness initiatives should be integrated into NICU practice models. Incorporating protected sleep periods within long or extended shifts may further mitigate fatigue-related impairment. Establishing a culture that prioritizes rest, resilience, and professional well-being is crucial to ensuring that neonatologists can continue providing high-quality care without the detrimental effects of chronic fatigue.

## Conclusion

Addressing shift-related fatigue is essential for neonatologist wellness and patient safety. As the workforce continues to evolve, with a growing proportion of female neonatologists [35], additional research is needed to guide sustainable scheduling solutions. Our findings emphasize the need for balanced scheduling policies that prioritize both clinician health and patient safety through evidence-based strategies.

## Supplementary information

Neonatologist Survey

## Data Availability

The data analyzed in this study were derived from a national survey of U.S. neonatologists. Individual survey responses are not publicly available due to confidentiality concerns, but de-identified data may be made available from the corresponding author upon reasonable request.
