# Supplementary material for: Impact of shift durations on sleep, fatigue, and wellness among neonatologists: a cross-sectional survey analysis
Source: J Perinatol. 2025 Apr 25;45(5):589–94. doi: 10.1038/s41372-025-02310-4 (PMC12222019; doi:10.1038/s41372-025-02310-4)
Supplement: Supplementary file 1 — Neonatologist Survey [file 41372_2025_2310_MOESM1_ESM.pdf]

Thank you for your interest in our research study regarding shift durations for neonatologists and the impact on sleep, safety, and wellness being conducted by Dr. Ryan McAdams, Dr. Renate Savich, Dr. Lily Lou, Dr. Patrick McNamara, and Dr. Satyan Lakshminrusimha. This questionnaire will help us understand the experiences of neonatologists regarding shift lengths in the neonatal intensive care unit (NICU) and obtain perspectives on potentially limiting shift durations. We invite you to participate in this study because you were part of a Listserv for neonatologists. Your feedback is important to help guide future discussions about optimal neonatology shift durations in the NICU.

Participation in the study consists of completing this questionnaire, which should take about 10 minutes. Your participation is voluntary, and you may skip any questions that you do not want to answer. Even if you start the questionnaire, you are not required to complete it. You can stop at any time by simply closing your web browser. The survey is confidential, and no one will be able to link your answers back to you. Please do not include your name or other information that could be used to identify you in the survey responses.

There are minimal risks to participants. The risk of a confidentiality breach will be minimized by storing survey data securely according to campus policy; by masking participant identities in publication; and when necessary, storing identifiable or sensitive information separately from other study data. We don't expect any direct benefits to you from participation in this study.

If you have questions about the study, please feel free to contact University of Wisconsin – Madison researcher Dr. Ryan McAdams at mcadams@pediatrics.wisc.edu. If you have questions about the survey, you may contact University of Wisconsin Survey Center Project Director, Griselle Sanchez-Diettert, at 608-262-2164 or gsanchez@ssc.wisc.edu

If you are not satisfied with the response of the research team, have more questions, or want to talk with someone about your rights as a research participant, please contact the University of Wisconsin confidential research compliance line at 1-833-652-2506.

By clicking "Next" below to start the survey, you indicate that you have read the information, are a neonatologist, and voluntarily consent to participate in this study. You also consent to the use of your de-identified responses for research purposes. Please feel free to print a copy of this page for your records.

Instructions to complete the survey:

To choose a response, click on the button that corresponds to your answer.

If you would like to change your answer, click on a different button, or for questions with multiple answers just click the same button again.

You may go back to earlier pages by clicking the 'BACK' button at the bottom of the screen. To advance to the next page of the survey click the 'NEXT' button.

If you start the survey and are unable to finish it, you can exit by simply closing the browser window and return to it later by clicking again on the link in your email. This will take you back to the next unanswered question.

After the entire survey has been completed, please click on the 'SUBMIT' button on the last page.

Once you click 'SUBMIT' you will not be able to re-enter the survey.

1. We begin with some questions about your shifts in the NICU.

In the past month, how often did you feel fatigued or sleepy during your work shifts in the NICU?

○ Never
○ Rarely
○ Sometimes
○ Very often
○ Extremely often

2. In the past month, how many hours was your typical <u>day</u> work shift in the NICU?

[ ] Hours

[programmer note, please limit numeric input to 0-24]

3. In the past month, how many hours was your typical <u>night</u> work shift in the NICU?

[ ] Hours

[programmer note, please limit numeric input to 0-24]

4. In the past month, was your typical NICU night shift in the hospital or from home?

○ In the hospital
○ From home

[Programmer note: display only if "From home" selected at previous question]

5. In the past month, when working night shifts from home, about how many times per night were you called by the NICU team to go to the hospital?

[ ] Times per night

[programmer note, please limit numeric input to 0-10]

6. In the past month, how many hours was the longest shift you worked at the NICU?

[ ] Hours

[programmer note, please limit numeric input to 8-60]

7. Do the following concern you about capping work shifts at a maximum of 12 hours?

	Yes	No
**a.** It would lead to working too many extra days of service	○	○
**b.** It would lead to working too many weekends	○	○

**c.** It would lead to poor patient care	○	○
**d.** It would prevent good team dynamics	○	○
**e.** It would hinder your personal protected time	○	○

8. In your opinion, what NICU shift length optimizes <u>patient safety</u>?

[ ] Hours

[programmer note, please limit numeric input to 0-60]

9. In your opinion, what NICU shift length optimizes <u>neonatologist wellness</u>?

[ ] Hours

[programmer note, please limit numeric input to 0-60]

10. In the past month, about how many times did you get less than 7 hours of sleep in a 24-hour period?

[ ] Times

[programmer note, please limit numeric input to 0-30]

11. In the past 6 months, how often did your fatigue or lack of sleep contribute to significant medical errors or near misses?

○ Never
○ Rarely
○ Sometimes
○ Very often
○ Extremely often

12. In your opinion, how much do shift durations over 16 hours…

	Not at all	A little	Somewhat	Quite a bit	A great deal
**a.** …increase risks to patient safety?	○	○	○	○	○
**b.** …negatively impact neonatologists' well-being?	○	○	○	○	○
**c.** …lead to a decrease in the quality of care provided?	○	○	○	○	○

13. Over the past month, outside of your scheduled NICU shifts, how many additional hours did you dedicate to NICU-related tasks, including EHR documentation?

[ ] Hours over the past month

[programmer note, please limit numeric input to 0-120]

14. How often do you use all your sick leave and vacation time allowed in a year?

- ○ Never
- ○ Rarely
- ○ Sometimes
- ○ Very often
- ○ Extremely often

15. Finally, we ask some questions about you.

What is your age?

[ ] Age

[programmer note, please limit numeric input to 25-80]

16. **What is your gender?**

- ○ Man
- ○ Woman
- ○ Non-binary
- ○ Not listed, please tell us: [ ]

17. How many years have you been practicing as a neonatologist?

[ ] Years

[programmer note, please limit numeric input to 0-60]

18. Which of the following best describes the setting you practice in most often?

- ○ University setting
- ○ Non-university setting

19. Do you work in each of the following NICU levels?

	Yes	No
**a.** Level 2	○	○
**b.** Level 3	○	○
**c.** Level 4	○	○

20. Do you have a sleep disorder, such as insomnia?

- ○ Yes
- ○ No

SUBMIT SCREEN

These are all the questions we have for you at this time. After the entire survey has been completed and you are ready to submit your answers, please click on the 'SUBMIT' button below. Once you click 'SUBMIT' you will not be able to re-enter the survey. Thank you for your participation!

<SUBMIT>

THANK YOU SCREEN

Thank you for your time! Your responses will help guide discussions about optimal shift durations for neonatologists.
